# Isolation of megakaryocytes using magnetic cell separation and adverse effects induced by diclofenac toxicity in an experiment

**DOI:** 10.25122/jml-2020-0129

**Published:** 2022-01

**Authors:** Irina Yuriivna Bagmut, Olexiy Sergiyovych Ivanov, Michael Ivanovich Sheremet, Sergiy Mykolayovych Smirnov, Igor Leonidovich Kolisnyk, Julia Viktorivna Ivanova, Mykhailo Yevgenievich Tymchenko, Vyacheslav Oleksievich Lazirskiy

**Affiliations:** 1. Department of Clinical Pathophysiology, Topographic Anatomy and Operative Surgery, Kharkiv Medical Academy of Postgraduate Education, Kharkiv, Ukraine; 2. Surgery Department No1, Bukovinian State Medical University, Chernivtsi, Ukraine; 3. Department of Medical Biology, Histology and Cytology, Lugansk State Medical University, Rubizne, Ukraine; 4. Zaytsev V.T. Institute of General and Urgent Surgery of National Academy of Medical Sciences of Ukraine, Kharkiv, Ukraine; 5. Department of Comprehensive Programming for the Development of Urgent Surgery and Intellectual Property Protection, SI ZIGUS NAMSU, Kharkiv, Ukraine; 6. Department of Surgery No. 1, Kharkiv National Medical University, Kharkiv, Ukraine

**Keywords:** megakaryocyte, Diclofenac, cytotoxic, mice models

## Abstract

This study investigates the response of bone marrow (particularly megakaryocytes) in mice under the influence of diclofenac sodium for 10 days using intraperitoneal injection at various doses. A fundamentally new immunomagnetic separation method was applied during the experiment, which helped obtain pure lines of bone marrow cells, particularly megakaryocytes (MC), without admixtures of other cells or their particles. The resulting cells completely retain their structure and can be used in further research. The study determined that different doses of diclofenac sodium have different effects on different groups of diabetes mellitus cells CD34-megakaryocytes. The use of 1.0 mg/ml sharply negatively affects the state of early populations of megakaryocytes (decrease by 80%, p=0.05), a dose of 0.025 mg/ml had the least effect on this population of cells (22.8%, p=0.05). The greatest number of average forms of diabetes mellitus 34 was observed when using a dose of 0.95 mg/ml (22.8%, p=0.05), with a gradual decrease in the dose, the indicator of this group of cells decreased. A dose of 0.03 mg/ml did not affect the quantitative state of megakaryocytes, and a dose of 0.025 mg/ml caused a slight decrease (16.6%, p=0.05). Indicators of mature cells of megakaryocytes CD 34- decreased in all studied groups, however, their maximum value reached a maximum decrease by 0.25 mg/ml (55.2%, p=0.05), the dose of diclofenac sodium 0.03 mg/ml, lower (18.4%, p=0.05). Diclofenac sodium in different doses has different effects on the degree of differentiation of CD 34-. Its introduction positively affects the state of intermediate forms of megakaryocytes, except for minimal doses, while the effect on early and mature forms in all cases turned out to be negative.

## Introduction

The formation process of megakaryocytes originates from precursors, which further differentiate into more mature forms due to the acquisition of glycoproteins and polyploidization, which is characteristic of mature forms of megakaryocytes. The process of polyploidization of megakaryocytes (as well as endomitosis or endoreduplication) ensures nuclear division without subsequent division of the cytoplasm. The ongoing processes provide a constant number of platelets in the blood and the formation of new forms of cells that, in a mature (polyploid) state, can produce platelets [1–3]. After carrying out an experiment using diclofenac sodium or any other substance in vivo, it is necessary to further investigate the result obtained.

There are many methods for isolating megakaryocytes from the bone marrow; however, those that preserve the largest possible number of viable and undamaged cells are the priority. Megakaryocytes from human bone marrow or guinea pigs are isolated by density gradient centrifugation. An alternative technique for obtaining a pure population of megakaryocytes is a countercurrent centrifugal illustration. These techniques make it possible to obtain mature and viable megakaryocytes with varying degrees of contamination with other bone marrow cells. In this regard, not all cells can be used in further molecular or biochemical studies. Methods for isolating pure cells based on fluorescence-activated cell sorting (FACS) allow obtaining the maximum number of pure and viable megakaryocytes that can be used for further research. However, this method is not always available [4–6]. To solve the problem of obtaining a pure population of megakaryocyte cells with maximum viability, suitable for further research, the method of immunomagnetic separation is used.

Immunomagnetic particles and monoclonal antibodies against cell surface antigen have been used recently to remove or collect surface antigen-specific cells. This method is sensitive and rapid for analyzing rare cells, even in complex cell mixtures, e.g., bone marrow, especially if they are influenced by medicinal or toxic drugs [7–10]. With pain syndromes of various specialties, non-steroidal anti-inflammatory drugs (NSAIDs) or narcotic analgesics are often used, particularly the drugs of opioid series. Traditionally, two NSAID groups are distinguished, those that affect COX-1 and COX-2, and selective COX-2 inhibitors, which are most preferred in treating pain syndrome, primarily caused by inflammation. The impact of the NSAID on COF-2 is reduced to the fact that they can inhibit the cycle of the conversion of arachidonic acid, which provokes further development of the inflammatory process. However, it was established that the uncontrolled and long-term reception of the NSAID can adversely affect the state of the gastrointestinal tract and the liver and kidney, blood coagulation processes. To treat inflammation, doctors successfully use many drugs, such as ibuprofen, naproxen, xefocam. However, the first drug from the NSAID group is diclofenac, which has a pronounced anti-inflammatory effect achieved by inhibiting prostaglandin synthesis [12–16].

In this paper, we describe a simple method for isolating pure populations of intact megakaryocytes, based upon their specific recognition by monoclonal antibodies coupled to magnetic particles and toxicity of diclofenac effects on the cell culture in an experiment.

## Material and Methods

### Study population

Wild-type littermate males were used for comparison. Expansion of the mouse colony was performed at Kharkiv Medical Academy of Postgraduate Education. The mice were held under typical laboratory conditions (temperature 22°C±2°C; 12h light–12h dark cycle and kept in plastic cages with free access to the commercial basal food and water. All mice were injected intraperitoneally with sodium diclofenac solution. All mice were divided into groups of 8 animals, with a total of 48 animals. A group of 8 animals was injected intraperitoneally with diclofenac sodium at a dose of 1.0 mg/ml, the second received 0.95 mg/ml, the third received 0.30 mg/ml, and the fourth received 0.25 mg/ml, the fifth group received diclofenac sodium at a dose 0.03 mg/ml, and the sixth 0.025 mg/ml for 10 days. The control group received 1 ml isotonic sodium chloride solution intraperitoneally. At the end of treatment, three mice from each group were euthanized by cervical dislocation at a fasting state.

### Bone Marrow Preparation

Mice were euthanized by cervical dislocation and their femurs removed. The marrow was collected by flushing each femur with 1 ml of ice-cold phosphate-buffered saline (PBS) with 1% bovine serum albumin (BSA) at mouse serum isotonicity. Marrow for each gradient was pooled from the femurs of three mice, gently monodispersed through a 25-gauge needle, and kept on ice. 25 aliquots of this cell suspension were counted on an electronic particle counter (EMD Millipore, Scepter Sensors) in a solution (30 mg/mI) of the cytoplasmic agent cetrimide to obtain the total nucleated cell count. After retrieval, the total number of bone marrow cells was counted. The number of nucleated cells was 2.5x10^8^ in 1 ml of pre-washed buffer.

### Magnetic Activated Cell Sorting

To obtain pure groups of cells suitable for analysis, we used the method of immunomagnetic separation, for which we used the CD140a (PDGFR-alpha) MicroBead Kit for mouse manufactured by Miltenyi Biotec, Germany, using paramagnetic beads (microspheres) coated with monoclonal unconjugated primary antibodies against the surface antigen of mouse megakaryocytes. The diameter of the spheres was 50 nm; they were not visualized by light microscopy and were completely biodegradable, while they did not damage the cell structure and did not affect the results of the study [9, 10, 16]. As a result, 3.0x10^4^ cells of CD34- megakaryocytes were obtained, which retained their viability and are completely ready for further research.

### Bone marrow cell counting

Femur bones were aseptically removed and placed individually in a 60 mm×15 mm petri dish with PBS. Tibia and femur were extracted aseptically, and the bone marrow (BM) cells were flushed from the tibia and femur of mice. BM cell suspension was subjected to ACK buffer for RBC depletion and washed and diluted in PBS. Cell viability and count analysis was made by the trypan blue exclusion method using a hemocytometer.

### Statistical analysis

The data were expressed as mean±standard error of the mean. Statistical differences among the prospective groups and their counterparts were analyzed using one-way analysis of variance (ANOVA) as part of an SPSS software package (v.16.0 for Windows, 2007; SPSS, Inc., Chicago, IL) by a post hoc test followed by Dunnett’s for several comparison tests to compare treated groups against respective controls. Significant differences were indicated by p values <0.05.

## Results

**Figure 1.**
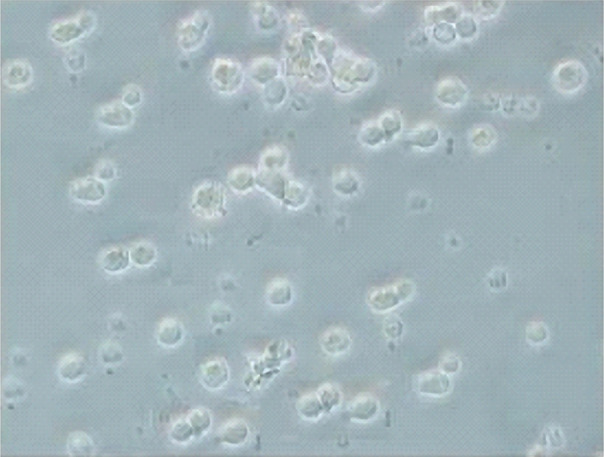
The characteristic morphology of the isolation of pure populations of intact megakaryocytes. Images were taken with an inverted microscope, 10X objective.

Megakaryocytes are difficult to isolate because of their fragility, tendency to aggregate, and varying sizes. An immunomagnetic cell sorting method has been developed to isolate intact human and mice megakaryocytes from whole bone marrow with a high purification and high recovery yield [10, 17, 18]. The results of the isolation method of pure populations of intact megakaryocytes (MK), based upon their specific recognition by monoclonal antibodies coupled to magnetic particles, are presented in Figure 1. The MKs were identified based on their large size, expression of platelet-associated antigens, and DNA ploidy levels. The median MK recovery was 12% of the original number of MKs. The median fraction of MKs among all nucleated cells after filtration was 53%. The MK viability after filtration was near 98%.

Microscopic examination confirmed that most isolated cells were megakaryocytes, most of which were large and had a well-preserved structure. Megakaryocytic viability was estimated to be greater than 90% in all samples. After being convinced of the complete viability of the cells, an analysis of the dependence of the differentiation of CD34 megakaryocytes on the dose of sodium diclofenac used was performed. All cells were divided into three groups: early, intermediate, and mature. The number of cells was counted in Goryaev’s cell. A clear relationship was established between the dose of sodium diclofenac used and the degree of differentiation of various megakaryocytes. So, the greatest changes took place among the early and mature forms of cells, the number of which decreased to one degree or another. In contrast, diclofenac sodium increased the number of medium forms of CD34- megakaryocytes (Table 1).

**Table 1.** The state of megakaryocytes CD34-bone pulp of mice after exposure to sodium diclofenac for 10 days (n=8; p=0.05).

**Dose (mg/ml)**	**Early**	**Intermediate**	**Mature**
**1.0**	7	70	23
**0.95**	9	71	20
**Control**	35	36	38

**Figure 2.**
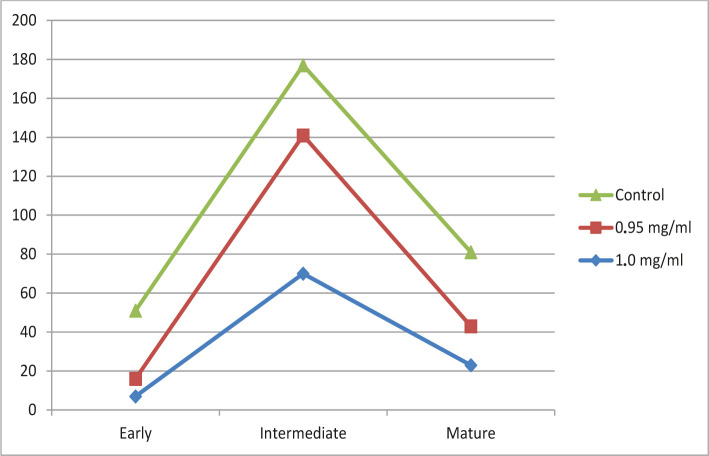
Change of the indicator of early, intermediate, and mature forms of megakaryocytes under the influence of sodium diclofenac in an amount of 1 and 0.95 mg/ml.

As shown in Table 1, there is a significant increase in the indicators of the average forms of megakaryocytes CD34- for the rest of the diclofenac sodium toxic in one form or another. So the dose of 1.0 mg/ml caused a decrease in the number of early forms of CD34- by 80% (p=0.05), while the indicator of intermediate forms increased by 194.4% (p=0.05), but the number of mature cells decreased by 39.5% (p=0.05) but not to the same extent as early forms of megakaryocytes. A dose of 0.95 mg/ml of diclofenac sodium gave approximately the same result. The indicator of early cell forms also decreased, but by 74.2% (p=0.05), the number of intermediate forms of CD34 – by almost 200% (p=0.05), which may indicate a positive effect of this dose. The indicator of mature cells decreased by 47.3% (p=0.05) compared to the control group (Figure 2).

Almost the same reaction was observed on the use of diclofenac sodium at a dose of 0.30 mg/ml and 0.25 mg/ml. However, inhibition of all cell forms occurred to a lesser extent than in the previous case (Table 2).

**Table 2.** The state of megakaryocytes CD34-bone pulp of mice after exposure to sodium diclofenac for 10 days (n=8, p=0.05).

**Dose (mg/ml)**	**Early**	**Intermediate**	**Mature**
**0.30**	11	52	23
**0.25**	15	62	17
**Control**	35	36	38

**Figure 3.**
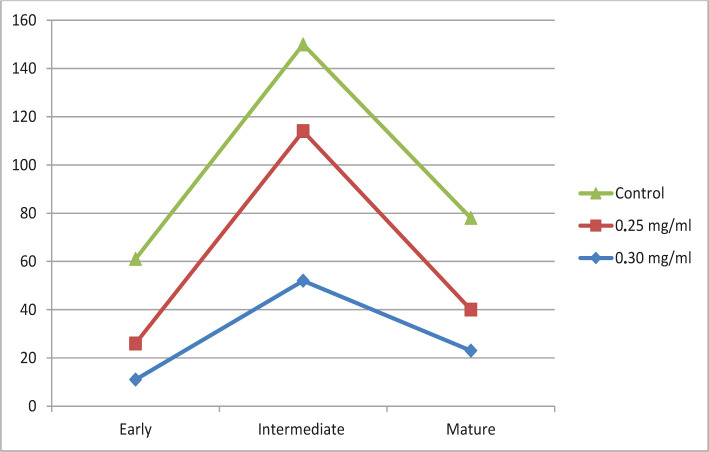
Change of the indicator of early, intermediate, and mature forms of megakaryocytes under the influence of sodium diclofenac in an amount of 0.30 and 0.25 mg/ml.

As shown in Table 2, the use of diclofenac sodium at a dose of 0.30 led to a decrease in early forms of CD34- by 68.5% (p=0.05), while the number of intermediate forms increased by 144.4% (p=0.05), but not when using 1.0 and 0.95 mg/ml diclofenac sodium. The indicator of mature forms of CD34- decreased compared to the control group by 39.4% (p=0.05). With the introduction of 0.25 mg/ml of diclofenac sodium into the body, the indicator of early cell forms decreased by 57.1% (p=0.05), while the indicators of intermediate forms increased by 127.7% (p=0.05), inverse the trend is traced with mature cells, the number of which decreased by 55.2% (p=0.05) (Figure 3).

Virtually there was no effect on CD34-diclofenac sodium in amounts of 0.03 and 0.025 mg/ml. Cell indices were practically the same as in the control group, indicating the minimal effect of the active chemical substrate (Table 3).

**Table 3.** The state of megakaryocytes CD34-bone pulp of mice after exposure to sodium diclofenac for 10 days (n=8, p=0.05)

**Dose (mg/ml)**	**Early**	**Intermediate**	**Mature**
**0.03**	25	36	31
**0.025**	27	30	28
**Control**	35	36	38

**Figure 4.**
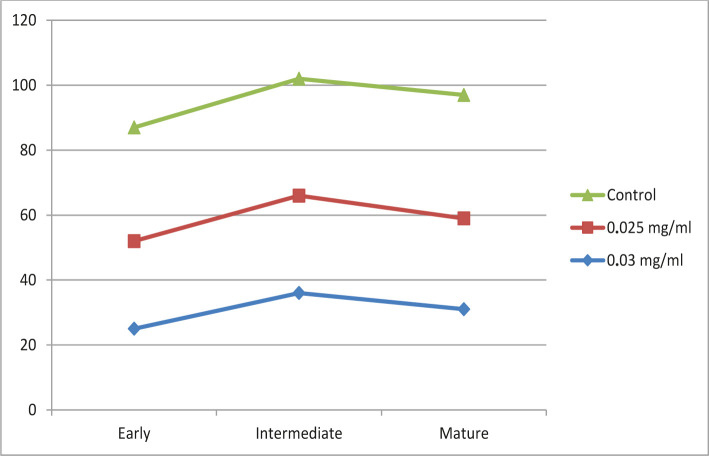
Change of the indicator of early, intermediate, and mature forms of megakaryocytes under the influence of sodium diclofenac in an amount of 0.03 and 0.025 mg/ml.

Thus, it was found that the introduction of 0.03 mg/ml diclofenac sodium into the body leads, although not so significantly, to a decrease in the indicators of early forms of CD34-bone marrow by 28.5% (p=0.05). At the same time, intermediate cells do not change (0%, (p=0.05)) compared to the control group, making it possible to assume the absence of influence on this group of cells of the given dose. The indicator of mature forms of megakaryocytes also decreased slightly by 18.4% (p=0.05). The minimum effect on the state of early forms of megakaryocytes was exerted by a dose of 0.025 mg/ml – 22.8% (p=0.05). The number of intermediate forms also decreased by 16.6% (p=0.05), which indicates some toxic effects of low doses of sodium diclofenac on them. The indicator of mature forms of CD34- decreased by 26.3% (p=0.05) after introducing 0.025 mg/ml diclofenac sodium into the animal’s body (Figure 4).

## Discussion

The study showed the toxicity of diclofenac sodium for early and mature forms of CD34-megakaryocytes at various doses. Increasing the dose leads to an increase in the average forms of megakaryocytes. Reducing the dose contributes to an increase in early and mature forms of megakaryocytes and a slight decrease in the intermediate forms. Small doses of diclofenac sodium can inhibit the inflammatory process with minimal impact on the state of bone marrow megakaryocytes, which can serve as a recommendation for further practical use. The prospect for further research is to study the effect of drugs on the state of bone marrow megakaryocytes depending on the dose of the active substrate used and the effect of diclofenac sodium on the state of other bone marrow cells depending on the dose.

## Conclusion

The effect of different doses of diclofenac sodium, administered intraperitoneally to mice at different concentrations, becomes apparent. Thus, the greatest toxic effect on early forms of CD34- was observed with the introduction of 1.0 mg/ml diclofenac sodium, which fell as the dose decreased. Increasing the dose had a beneficial effect on the intermediate forms of CD34-, where the maximum effect was observed with 0.95 mg/ml of diclofenac sodium. A decrease in the dose to 0.03 mg/ml led to a neutral effect, and a further decrease to 0.025 mg/ml caused a decrease in intermediate CD34-, which may indicate some toxic effect of this dose. The dose of 0.25 mg/ml turned out to be the most toxic for mature forms of megakaryocytes since the indicator decreased by 55.2% compared to the control group. As the dose was increased or decreased, mature CD34-values decreased to varying degrees.

## Acknowledgments

### Conflict of interest

The authors declare no conflict of interest.

### Ethics approval

The study was approved by the Kharkiv Medical Postgraduate Academy Animal Care and Use, protocol 2 of 26.06.2020.

### Personal thanks

The authors are enormously grateful to the leadership of the Kharkiv Medical Academy of Postgraduate Education for help in conducting the research.

### Authorship

IYB contributed to the concept of the article, developed the purpose and objectives of the study. OSI contributed to the research methodology and participated in the experimental part. MIS contributed to writing the original draft and editing the text. SMS contributed to the data processing and editing of the original text. ILK contributed to the data processing and experimental part of the study. JVI contributed to data analysis, MYT contributed to data processing and analysis. VOL contributed to the experiment and data analysis.
